# Cooperative Rotation and Spin State Switching of Molecules in Artificial Arrays

**DOI:** 10.1021/acs.jpclett.5c03962

**Published:** 2026-01-27

**Authors:** Niklas Ide, Arnab Banerjee, Alexander Weismann, Richard Berndt

**Affiliations:** Institut für Experimentelle und Angewandte Physik, Christian-Albrechts-Universität, 24098 Kiel, Germany

## Abstract

Artificial arrays of tin phthalocyanine molecules have been studied on Pb(100) using scanning tunneling microscopy (STM). The molecules adopt two azimuthal orientations with distinct spin states, which results in a checkerboard pattern in STM images. Upon converting the central molecule by current injection from its pristine state with the Sn ion above the molecular plane to a conformation with Sn between the macrocycle and the substrate, the orientations of all molecules are swapped. This cooperative rotation is modeled by combining the potential energies of the intermolecular and the molecule–substrate interactions.

## Graphical abstract

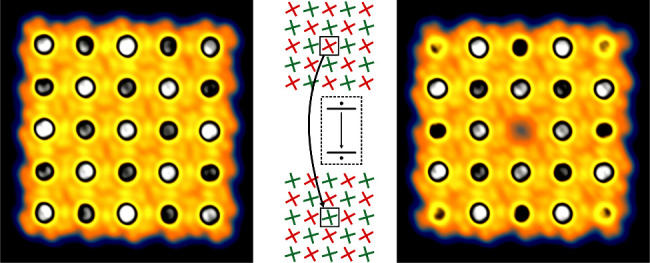

Conformational changes of molecules are important for biological and man-made molecular machines. Significant efforts have been made to implement simple molecular devices on surfaces and experiments have demonstrated switching of individual molecules induced with the scanning tunneling microscope (STM).^1–4^ In particular, isolated molecular rotors have been studied on surfaces.^5–19^

Cooperative effects, i.e., the transduction of a conformational change of an adsorbed molecule to its neighbors, have not often been reported. Light-induced cooperativity was observed from monolayers of azobenzene derivatives^20–23^ Using a STM, the propagation of tautomerization over one intervening molecule has been achieved^24^ and geometrical isomerization has been remotely triggered in a binuclear complex.^25^ The sequential hopping of neighboring CO molecules similar to a chain of dominoes^26^ and rotations of arrangements of a few star-shaped molecules have been demonstrated.^27–29^ Extended structural modifications have been induced on semiconductor surfaces.^30,31^ Collective switching of the spins has been reported from Ni atoms connected by organic linkers a gold surface.^32,33^

Here we investigate artificial arrays arranged from tin-phthalocyanine (SnPc) on a Pb(100) surface at low temperature (4.2 K) in ultrahigh vacuum. Further experimental details are presented in the Supporting Information. The complex adsorbs planar to metal surfaces with the Sn ion above (Sn↑Pc) or below (Sn↓Pc) the macrocycle.^45^ We prepared square arrays of up to 25 Sn↑Pc complexes (Figure 1a) that exhibit a checkerboard pattern with two distinct molecular orientations α_1_ and α_2_. Upon switching the central molecule in 5 × 5 and 3 × 3 arrays from its Sn↑-state to Sn↓ by current injection, the orientations of all molecules change from α_1_ to α_2_ and vice versa. Simultaneously, the spin states of the molecules are exchanged. A model of the interactions among the molecules and with the substrate reproduces the observations.

**Figure 1:**
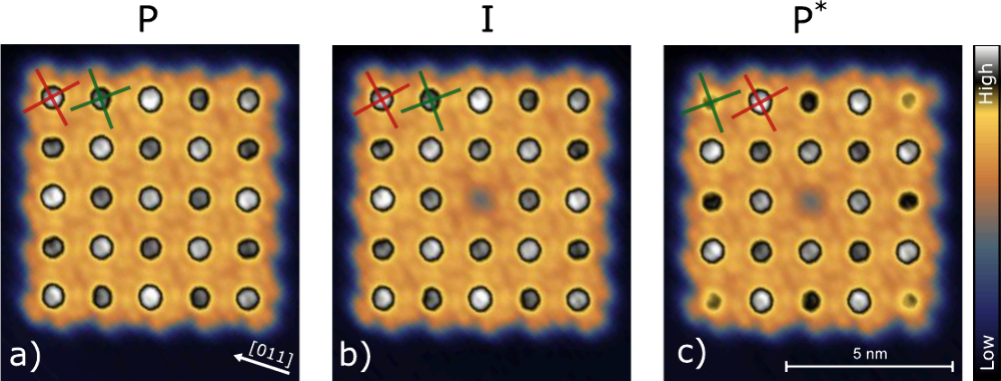
Topographs of a 5 × 5 array of Sn↑Pc complexes. (a) As-prepared state, denoted **P**. The molecules exhibit two nonequivalent molecular orientations that give rise to slightly different apparent heights (light and dark gray) and a checkerboard pattern with bright corner molecules. The [011] substrate direction is indicated by an arrow. Red and green crosses mark the orientations of the lobes of bright and dark molecules, respectively. (b) Intermediate state **I** observed after converting the central molecule from Sn↑ to Sn↓. All molecules except the central one are unchanged. The transition was induced by placing the tip over the central molecule and applying –1.7 V and 100 pA. (c) Final state P* imaged approximately 10 min later, after a spontaneous relaxation. The checkerboard pattern from panel (a) has been inverted. For example, corner molecules now appear dark. Imaging conditions: *I* = 50 pA, *V* = 20 mV. The color bar spans a height range of 310 pm.

Figure 1a shows a constant-current topograph of an array of 5 × 5 Sn↑Pc molecules prepared by lateral manipulation with the STM tip. The apparent height of the molecules varies with a checkerboard pattern. For example, the corner molecules appear higher than their nearest neighbors. In addition, the azimuthal orientations of the molecular lobes vary. The double lobe structure and its orientation is easily discernible at corner molecules, while the pattern in the interior of the array is rather complex. Close inspection reveals that the isoindole lobes enclose angles of α_1_ = 42° and α_2_ = 56° with the ⟨110⟩ directions of the substrate (Figure 1, red and green crosses, respectively; see also Supporting Information). This pattern was first found in self-assembled arrays^34^ and therefore appears to be the lowest-energy configuration. We denote it **P**. Closely related patterns were observed from PbPc,^35^ H_2_Pc,^36,37^ AlPc,^38,39^ and InPc^40^ on Pb(100). The twist between neighboring molecules is favorable, as it allows hydrogen bonding between C–H groups and aza-nitrogen atom of adjacent molecules.^39^ The resulting induced dipoles at C–H sites produce an alternating electrostatic potential between α_1_ and α_2_ molecules.^36,38^

The conversion from the Sn↑Pc to the Sn *↓* conformation was previously demonstrated and attributed to electron removal from the HOMO.^34,41,42^ Here this technique is used to selectively convert the central molecule of the 5 × 5 array. To this end, the tip was centered above the molecule and the sample voltage *V* was reduced below −1.6 V while keeping the current constant. This leads to an abrupt reduction of the tip height signaling the transition to Sn↓Pc. An image of this new state **I** is shown in Figure 1b. Except for the central molecule, the pattern is unchanged. After a delay of ≈10 min, all molecules of the array spontaneously changed their orientation from α_1_ to α_2_ and vice versa (Figure 1c) leading to the final state P*. We note that the transformation of the array is not reversible because the transition of the central SnPc molecule from Sn↑ to Sn↓ is irreversible.

The delay mentioned above was observed only in a single case. Usually, after inducing the transition at the center of the array, all molecules had already rotated when the subsequent image was recorded. Nevertheless, the data suggests that the state **I** is metastable.

To highlight the differences between states **P** and P*, Figure 2 shows the topographs of two corner molecules with an adjusted color scheme. There is a distinct difference between the orientations of the exterior lobes and the apparent height is changed.

**Figure 2:**
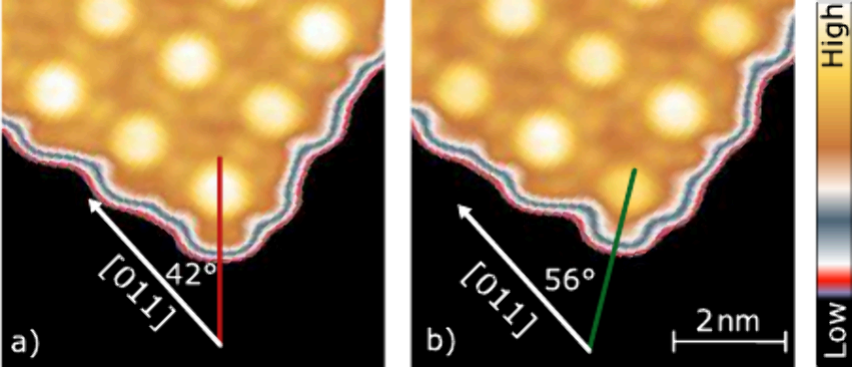
Corner of a 5 × 5 array. (a) Image of the original state **P**. (b) Image recorded after inducing a transition of the center molecule from Sn↑ to Sn↓ (state P*). Green and red lines indicate the orientation of isoindole lobes, which enclose angles of 56° and 42° with the [011] substrate direction.

The **P**-to-P* transition presented above was reproduced on several 5 × 5 arrays. We also induced the same effect on 3 × 3 arrays as shown in Figure 3. In the as-prepared array (Figure 3a), the checkerboard pattern of the orientations α_1_ and α_2_ does not lead to a clear contrast in the apparent heights (only the center molecule appears slightly higher), but it may be verified by inspecting the lobe orientations at the island edges. In particular, the four corner molecules exhibit α_1_. After switching the central molecule from Sn↑ to Sn↓, the corner molecules are reoriented to α_2_ and appear lower than the edge molecules, which underwent the opposite transition from α_2_ to α_1_.

**Figure 3:**
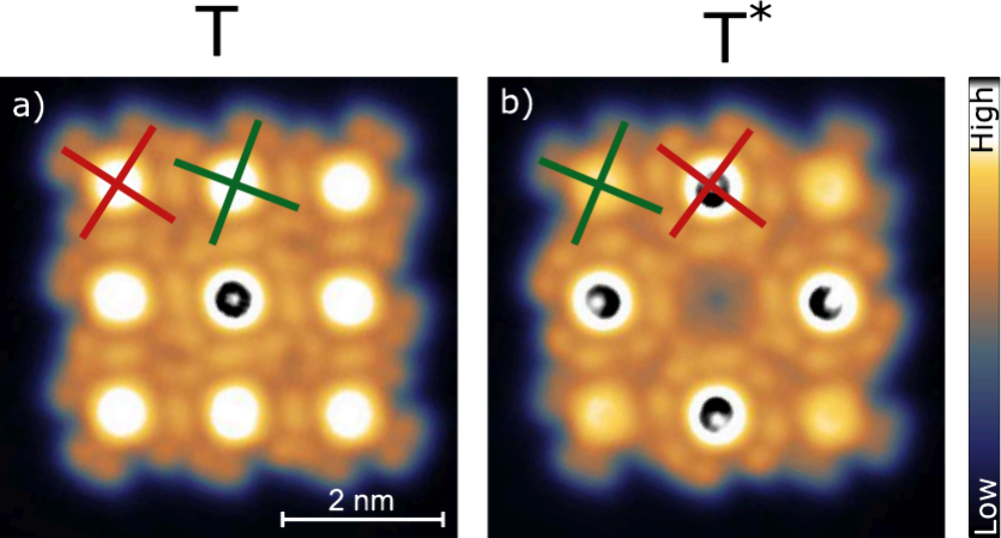
Topographs of an artificial 3 × 3 array comprised of Sn↑Pc complexes (*V* = 10 mV). The molecules exhibit alternating orientations α_1_ and α_2_. Examples are indicated by red and green crosses, respectively. (a) As-prepared state denoted **T**. (b) Image recorded after converting the central molecule from Sn↑ to Sn↓, denoted T*. The color bar spans a height range of 300 pm.

In monolayers, the different orientations of the SnPc complexes come along with different spin states.^34^ This effect also exists in the artificial arrays. Spectra of the differential conductance (d*I*/d*V*, Figure 4) recorded from the edge (green) and corner (red) molecules of a 3 × 3 cluster in the **T** state are distinctly different. The corner molecules (green) show the typical spectrum of superconducting Pb with two coherence peaks with identical heights. On the edge molecules (red), the peaks are shifted inward by ≈50 μV and their heights are no longer identical. This effect may be quantified by an asymmetry parameter χ defined as the ratio of the peak height difference and the sum of the heights, χ = (*A* ^+^ – *A* ^–^)/(*A* ^+^ + *A* ^–^), which amounts to ≈4% in this case. In the T* state, the spin signatures of the edge and corner molecules are interchanged. On edge molecules, the inward shift is ≈120 μV and χ ≈ 6% while spectrum of the corner molecules show symmetric coherence peaks at ±2.42 mV. The peak height asymmetries and the inward shift are hallmarks of a Yu-Shiba-Rusinov state,^43^ and signal the presence of a spin that is localized to corner molecules in the **T** state and to edge molecules in T*.

**Figure 4:**
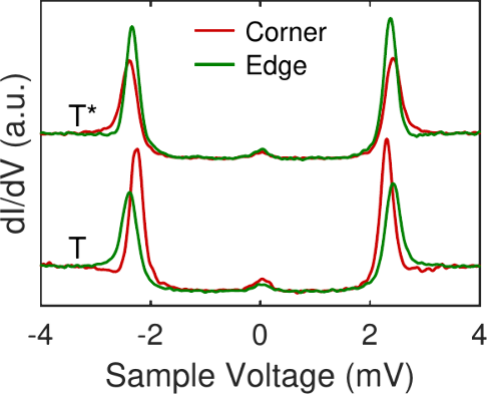
Exchange of spin states in a 3 × 3 cluster. Differential conductance spectra acquired on corner (red) and edge (green) molecules of the pristine (T, lower curves) and the switched (T*, upper curves) conformation. Yu-Shiba-Rusinov states indicating a molecular spin lead to peaks shifted into the superconductor gap. They are seen on corner (edge) molecules in T (T*), i.e. molecules with α_1_ orientation.

The fact that the transition of a single complex triggers a rotation of an entire array is remarkable. In the Supporting Information, we present a model that rationalizes this striking effect in terms of competing molecule–molecule and molecule–substrate interactions. The model is motivated by the observation that isolated Sn↑Pc molecules prefer azimuthal orientations in which their isoindole lobes form angles of 0 and 45° with the ⟨110⟩ directions of the Pb(100) substrate. In dense islands, however, the two molecules of a unit cell assume two distinct orientations α_1_ = 42° and α_2_ = 56°. The different angles of nearest neighbors imply a bistability, because they may swap their orientations.

Figure 5 summarizes modeling results. The intermolecular interaction energies $\Phi_{M}^{↑}$ (blue) and $\Phi_{M}^{↓}$ (red) in unswitched and switched arrays, respectively, are displayed in Figure 5a. For the switched array the central Sn↓Pc molecules is oriented at 42° in the **I** configuration and at 56° in the final P* state. The potentials are very similar and exhibit minima at α = 41° and 58° (Figure 5a), which are close to but not identical to α_1_ and α_2_. The minima are degenerate for the $\Phi_{M}^{↑}$ potential; however this degeneracy is lifted for the $\Phi_{M}^{↓}$ potential due to a small asymmetry of the interaction between Sn↑ and Sn↓ molecules analyzed in the Supporting Information. The barrier separating the minima, which impedes a coherent rotation, is ≈480 meV high. This is the sum of 40 pair interactions with a barrier height of ≈12 meV as determined in the case of an AlPc dimer.^39^ For 3 × 3 arrays the barrier height of the intermolecular interaction (144 meV) is reduced to a net barrier of 15 meV. Indeed, we never observed an intermediate state from 3 × 3 arrays. Larger arrays lead to increased barrier heights (Supporting Information).

**Figure 5:**
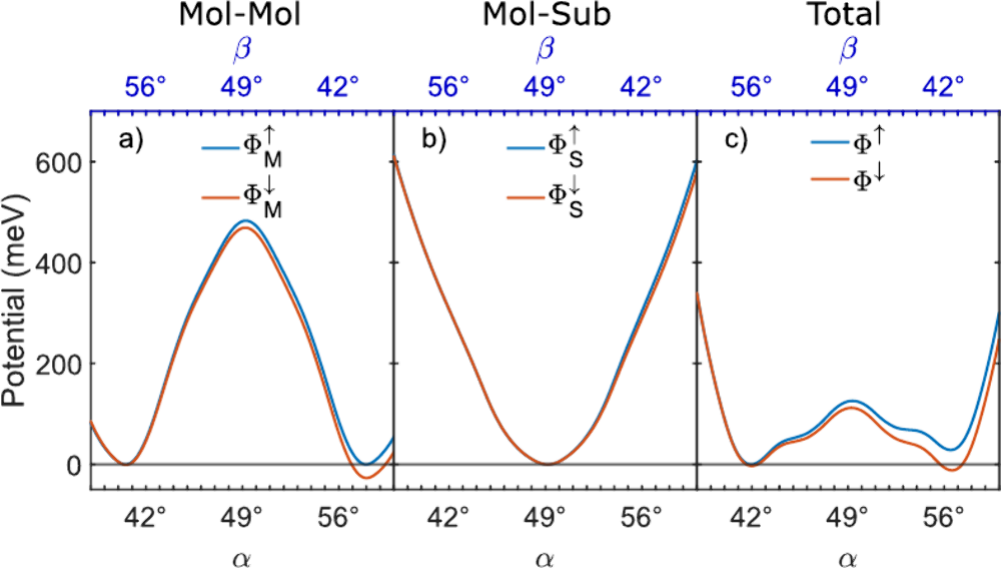
Potential energies of 5 × 5 array vs the azimuthal orientations α of the central and β neighboring molecules. Blue and red lines correspond to arrays with the central molecule in the Sn↑ and Sn↓ conformations, respectively. (a) Molecule–molecule interactions $\Phi_{M}^{↑}$ and $\Phi_{M}^{↓}$ (eqs (1) and (2) of the Supporting Information). (b) Molecule–substrate potential energies $\Phi_{S}^{↑}$ and $\Phi_{S}^{↓}$ according to eqs (4) and (5) of the Supporting Information. (c) Total potential energies $\Phi^{↑}=\Phi_{M}^{↑}+\Phi_{S}^{↑}$ (blue) and $\Phi^{↓}=\Phi_{M}^{↓}+\Phi_{S}^{↓}$ (red).

Figure 5b presents the molecule–substrate potentials $\Phi_{S}^{↑}$ and $\Phi_{S}^{↓}$ of arrays with central Sn↑ and Sn↓ molecules, which are sums over molecules with two alternating orientations (see Supporting Information) and exhibit a minimum at 49°. The total potential energies Φ^↑^ and Φ^*↓*^ obtained by adding the respective intermolecular and molecule–substrate interactions, are displayed as blue and red curves in Figure 5c. The double-well shape of the pair potential remains with the minima slightly shifted inward by ≈1°. More importantly, the rotation barrier is drastically reduced to ≈125 meV. In other words, the competition of intermolecular and molecule–substrate interactions leads to a fairly low barrier, despite the large number of molecules involved.

In summary, a cooperative rotation of all molecules in SnPc clusters and a concomitant change of the spin states has been observed upon inducing a conformational change of the central molecule. This surprising effect is enabled by a competition between the interactions of the molecules with the substrate and their neighbors. The competition results in bistable azimuthal orientations and a fairly low transition barrier between them. The intermolecular interaction is largely due to induced charges in H bonds that are controlled by the proximity of N atoms of a neighbor molecule. One may speculate that related effects may be used in molecular machines involving other hydrogen-bonded structures. Indeed, similar bistability was observed from azopyridine supramolecules on Au(111).^44^

## Supplementary Material



## Notes

Model used and information on the experimental and theoretical methods (PDF)
