# Supplementary material for: Cooperative Rotation and Spin State Switching of Molecules in Artificial Arrays
Source: J Phys Chem Lett. 2026 Jan 27;17(5):1378–82. doi: 10.1021/acs.jpclett.5c03962 (PMC12884518; doi:10.1021/acs.jpclett.5c03962)
Supplement: Supplementary file 1 [file jz5c03962_si_001.pdf]

Supporting Information for Cooperative Rotation and Spin State Switching of Molecules in Artificial Arrays

Niklas Ide, Arnab Banerjee, Alexander Weismann,* and Richard Berndt*

Institut für Experimentelle und Angewandte Physik, Christian-Albrechts-Universität, 24098 Kiel, Germany

E-mail: weismann@physik.uni-kiel.de; berndt@physik.uni-kiel.de

Methods

Experiments

The Pb(100) single crystal substrate was prepared by Ar-ion sputtering and annealing to ≈ 500 K. Submonolayer amounts of SnPc were deposited using a Knudsen cell. The Pb substrate was kept close to room temperature during deposition and then cooled to cryogenic temperature.The measurements were made with an ultrahigh vacuum (UHV) scanning tunneling microscope operated at $T = 4.2\,\mathrm{K}$. STM-tips were cut from W or Pb wire and sputter-cleaned in UHV. STM topographs were measured at constant currents of the order of 50 pA. Artificial molecular arrangements were constructed by laterally moving single molecules on the Pb surface employing $V = 4\,\mathrm{mV}$ and a current of ≈ 4 nA.

Calculations

Density functional theory calculations were performed for a slab of nine Pb(100) layers and two SnPc molecules per unit cell using the QuantumATK software.[1] The generalized-gradient approximation (GGA) was employed, van der Waals interactions were included using the Grimme DFT-D3 method, and the k-space was sampled on a 4×4×1 Monkhorst-Pack grid. The structure was optimized until the residual forces on all atoms were $< 0.05\,\mathrm{eV\AA^{-1}}$.

Geometry Notation

Figure 1 displays sketches of the conformations of the adsorbed molecules denoted (a) Sn↑Pc and (b) Sn↑Pc. In addition, the azimuthal angles $\alpha_1 = 42°$ and $\alpha_2 = 56°$ between an isoindole lobe and a ⟨110⟩ direction of the substrate are shown in (c) and (d).

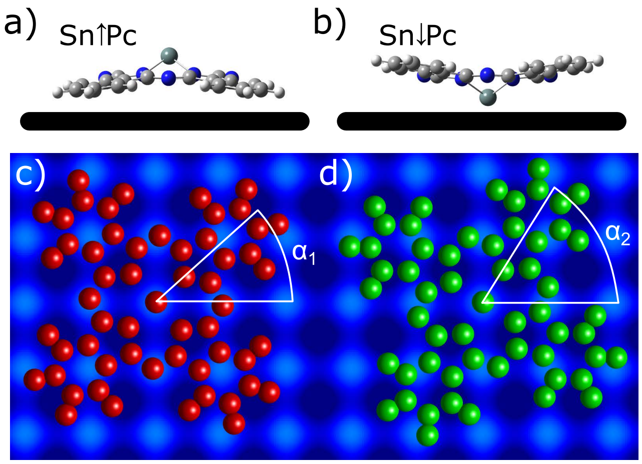

Figure 1: Naming scheme of the molecular conformations and the azimuthal angles. Spheres indicate the atoms of the SnPc molecules. The Pb substrate is represented by black bars in panels a and b and by a blurred blue lattice in c and d.

Model Potential

To estimate the energy barrier for the relaxation from **I** to $\mathbf{P}^*$ we constructed a potential energy based on molecule-molecule and molecule-substrate interactions. The intermolecular interaction has been evaluated in detail for AlPc on Pb(100) using experimental data of molecular dimers and corresponding density functional theory calculations.[2] Because the potential minima are mainly determined by the proximity of aza-nitrogen atoms and neighboring C–H groups, we expect the interaction potential between two SnPc molecules to be qualitatively similar.

Below, we denote the orientation of the central molecule as α and assume neighboring molecules to be rotated to the angle β with $\alpha+\beta=\alpha_1+\alpha_2$. This condition ensures that a clockwise rotation of a molecule is accompanied by an anticlockwise rotation of its neighbors. In the closely related case of AlPc, this was found to minimize the rotation barrier height.

The potential energy of the molecule-molecule interaction in a pristine 5×5 array (state **P**) can be written as

$$\Phi_{\mathrm{M}}^{\uparrow}(\alpha,\beta)=40\,\phi_{\mathrm{M}}^{\uparrow\uparrow}(\alpha,\beta). \tag{1}$$

Here $\phi_{\mathrm{M}}^{\uparrow\uparrow}$ is the interaction of a single pair of Sn↑Pc molecules. In the 5×5 arrays, 40 pair bonds between Sn↑Pc molecules are present. When the central molecule is in the Sn↓ state (arrays **I** and $\mathbf{P}^*$), the sum of the pairwise interactions is modified:

$$\Phi_{\mathrm{M}}^{\downarrow}(\alpha,\beta)=36\,\phi_{\mathrm{M}}^{\uparrow\uparrow}(\alpha,\beta)+4\,\phi_{\mathrm{M}}^{\uparrow\downarrow}(\alpha,\beta). \tag{2}$$

4 pair interactions among Sn↑Pc molecules are substituted by interactions in Sn↑/Sn↓ pairs.

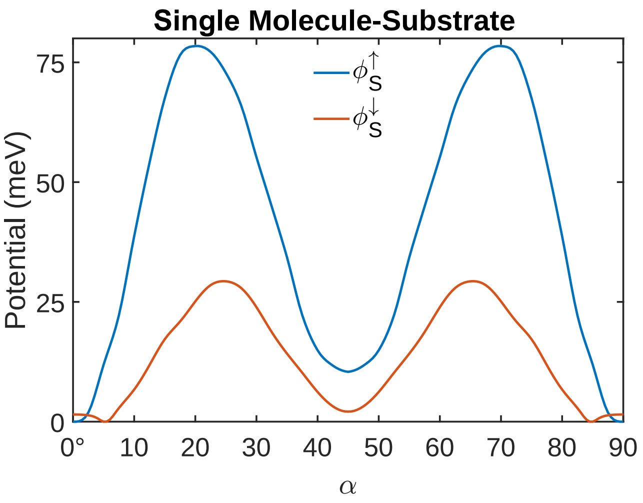

Figure 2: Potential of the interaction of isolated Sn↑ and Sn↓ molecules on top sites with the substrate from Ref. 3.

While the pair-interaction between Sn↑Pc molecules is symmetric, $\phi_M^{\uparrow\uparrow}(\alpha,\beta)=\phi_M^{\uparrow\uparrow}(\beta,\alpha)$, for a pair of Sn↑Pc and Sn↓Pc molecules is not. The difference, evaluated for the optimal orientations α_1 and α_2, is denoted δ:

$$\delta=4\left(\phi_{\mathrm{M}}^{\uparrow\downarrow}(\alpha_1,\alpha_2)-\phi_{\mathrm{M}}^{\uparrow\downarrow}(\alpha_2,\alpha_1)\right). \tag{3}$$

The molecule-substrate interaction is calculated from the corresponding potentials of isolated SnPc molecules $\phi_{\mathrm{S}}^{Sn\uparrow}$ and $\phi_{\mathrm{S}}^{Sn\downarrow}$ determined in Ref. 3 (Figure 2). In state **P** (only Sn↑Pc molecules), the array contains 13 (12) molecules with the orientation α (β). For the molecule-substrate potential we thus use

$$\Phi_{\mathrm{S}}^{\uparrow}(\alpha,\beta)=13\phi_{\mathrm{S}}^{\uparrow}(\alpha)+12\phi_{\mathrm{S}}^{\uparrow}(\beta) \tag{4}$$

In states **I** and $\mathbf{P}^*$, a Sn↓Pc molecule substitutes one Sn↑Pc:

$$\Phi_{\mathrm{S}}^{\downarrow}(\alpha,\beta)=12\phi_{\mathrm{S}}^{\uparrow}(\alpha)+12\phi_{\mathrm{S}}^{\uparrow}(\beta)+\phi_{\mathrm{S}}^{\downarrow}(\alpha) \tag{5}$$

The total potential Φ^x ($x=\uparrow,\downarrow$) is obtained by adding the respective components Φ_{M}^x and Φ_{S}^x.

In general, the intermolecular and the molecule substrate potentials of $n\times n$ arrays in the initial state (all molecules Sn↑) can be written as

$$\Phi_{\mathrm{M}}^{\uparrow}(\alpha,\beta)=2n(n-1)\,\phi_{\mathrm{M}}^{\uparrow\uparrow}(\alpha,\beta) \tag{6}$$

and

$$\Phi_{\mathrm{S}}^{\uparrow}(\alpha,\beta)=\frac{n^2+1}{2}\,\phi_{\mathrm{S}}^{\uparrow}(\alpha)+\frac{n^2-1}{2}\,\phi_{\mathrm{S}}^{\uparrow}(\beta), \tag{7}$$

n is an odd number. For the switched arrays minor changes can be expected. The total barrier height is estimated as

$$\Delta\approx 2n(n-1)\Delta_{\mathrm{M}}-n^2\Delta_{\mathrm{S}}. \tag{8}$$

$\Delta_{\mathrm{M}}=12\,\mathrm{meV}$ is the rotation barrier derived from the intermolecular interaction $\phi^{\uparrow\uparrow}(\alpha,\beta)$ and $\Delta_{\mathrm{S}}\approx 14\,\mathrm{meV}$ is the energy due to the molecule-substrate interaction per molecule for a rotation from 42° to 49° $\left(1/n^2\left[\Phi_{\mathrm{S}}^{\uparrow}(42^\circ,56^\circ)-\Phi_{\mathrm{S}}^{\uparrow}(49^\circ,49^\circ)\right]\right)$.

Estimation of δ

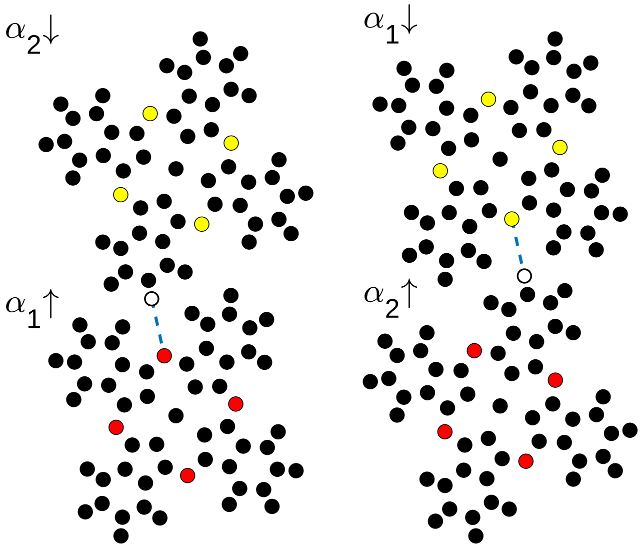

Figure 3: Sketches of the unit cells of the DFT calculations and relevant polarization charges. The cells are comprised of two SnPc molecules. All atoms are represented by black dots except for the aza-nitrogen atoms (yellow and red) and the H atom involved in bonding to the neighbor molecule (white). In both columns, the upper molecule models the center molecule of an 5×5 array after it has been switch to the Sn↓ state. The lower molecule is in the Sn↑ state and represents one of the four nearest neighbors. In the left column, the orientations of the upper and lower molecule are α_2 and α_1, respectively. In the right column, the orientations have been swapped. Dashed lines connect the H-N pair contributing most to the pair interaction. In the left column, which corresponds to state $\mathbf{P}^*$, the aza-nitrogen (red) is fairly negative, which implies strong bonding of the molecules. In the right column, which models the state **I**, the aza-nitrogen (yellow) is less negative (by 0.009 e), and the bond is weaker. This effect occurs with each of the four nearest neighbors.

In **P** and $\mathbf{P}^*$ islands, α_1 and α_2 are the respective majority orientations. To reproduce this experimental result, we use the fact that the total potential is highly sensitive to the energy difference δ. We find that $\delta < -27\,\text{meV}$ is required to obtain the observed majority orientations. When δ is decreased beyond $-400\,\text{meV}$, the barrier collapses and state **I** is no longer metastable.

To rationalize the sign of δ, DFT calculations of SnPc monolayers on Pb(100) were performed with unit cells containing a Sn↑Pc and a Sn↓Pc molecule (Figure 3). Two pairs of azimuthal orientations were considered, (α_2, α_1) and (α_1, α_2) for the Sn↓ and Sn↑ molecules that represent the central molecule of an **I** or $\mathbf{P}^*$array and one of it nearest neighbors. We find that the Mulliken charge of the aza-nitrogen atoms that mediate the bonding between the two molecules differ between the two cases. This leads to a contribution $\delta < 0$ to the binding energy that underpins the increased stability of $\mathbf{P}^*$ compared to **I**. We did not attempt to quantitatively determine δ because Mulliken charges depend on the basis set used.

References

1. Synopsys QuantumATK, version V-2023.12, www.synopsys.com/silicon/quantumatk.html.

2. Li, C.; Lu, Y.; Li, R.; Wang, L.; Weismann, A.; Berndt, R. Mechanically Interlocked Molecular Rotors on Pb(100). *Nano Lett.* **2025**, *25*, 1504–1511.

3. Banerjee, A.; Ide, N.; Lu, Y.; Berndt, R.; Weismann, A. Adsorption-Site- and Orientation-Dependent Magnetism of a Molecular Switch on Pb(100). *ACS Nano* **2025**, *19*, 7231–7238.
